# Evaluation of the diagnostic performance of a commercially available point-of-care test for post weaning diarrhoea in pigs-a pilot study

**DOI:** 10.1186/s40813-022-00292-9

**Published:** 2022-12-12

**Authors:** Nadia Jakobsen, Nicole Bakkegård Goecke, Ken Steen Pedersen

**Affiliations:** Department of Veterinary and Animal Sciences, Faculty of Health and Medical Sciences, University of Copenhagen, 1870 Frederiksberg C, Denmark

**Keywords:** Point-of-care, Rapid diagnostics, Post weaning diarrhoea, Enterotoxigenic *E. coli*, Rotavirus

## Abstract

**Background:**

Post weaning diarrhoea is expected to become an increasing problem in pig herds following the outphasing of medicinal Zinc Oxide. Currently, no equally effective substitute has been found and an increase in metaphylactic batch medication with antibiotics is expected. However, prudent use of antibiotics is needed to mitigate antibiotic resistance development and one option could be pre-treatment diagnostics. Employing a point-of-care test in a herd could provide fast diagnostics and help guide antibiotic treatment. Hence, the aim of this study was to evaluate the diagnostic performance of a commercially available point-of-care test for enterotoxigenic *Escherichia coli* (ETEC) F4, ETEC F18 and rotavirus in weaned pigs.

**Results:**

In total 115 diarrheic samples from two conventional herds were included in the evaluation of the Rainbow Piglet Scours test, which was compared to microbiological PCR analyses. The comparison yielded a diagnostic sensitivity, diagnostic specificity, positive and negative predictive value of 0.28, 0.99, 0.92 and 0.70 for ETEC F4, 0.40, 0.92, 0.91 and 0.45 for ETEC F18 and 0.67, 0.88, 0.91 and 0.61 for rotavirus.

**Conclusions:**

The point-of-care test yielded a low diagnostic sensitivity and a high diagnostic specificity for ETEC F4, ETEC F18 and rotavirus. Due to the high level of false negatives, the test cannot be recommended for individual diagnostics on pig-level.

## Introduction

Post weaning diarrhoea (PWD) is an enteric disease affecting pigs during the first weeks after weaning. The clinical symptoms of PWD are weight loss, diarrhoea and unthriftiness [1]. Post weaning diarrhoea is associated with economic losses due to use of medicines, depressed growth and mortality [2].

Currently, PWD is mitigated by medicinal Zinc Oxide (ZnO) to the feed during the first 14 days after weaning. A strategy that has been used in Denmark since the outphasing of antibiotic growth promoters. Using ZnO successfully controls PWD, but medicinal ZnO was banned in Europe from June 2022 [3], due to environmental risks caused by accumulation of Zn in terrestrial and aquatic environments and possible co-selection of antimicrobial resistance [4]. PWD can be prevented by management interventions and vaccination, but an equally cheap and easy replacement of ZnO has yet to be identified. Therefore, the ban of medicinal ZnO in pig feed is expected to cause an escalation in PWD cases.

The predominant treatment protocol for PWD in Denmark is based on yearly diagnostics and metaphylactic antibiotic batch medication. Studies on PWD, have revealed that the common aetiological agents of PWD are enterotoxigenic *Escherichia coli* (ETEC) F4 and F18 [5, 6] and rotavirus [7]. However, the disease is multifactorial and the clinical manifestation depend on interaction between factors related to pen environment, management, immunization, feed and pathogens [2].

In addition to virus infections, some cases are non-infectious [2] and in both cases antibiotic overuse may occur when metaphylactic batch medication is applied, which might exacerbate antimicrobial resistance (AMR). Antimicrobial resistance is a major health, public and political concern and multiresistance to several antibiotics therapeutically used for ETEC based PWD has been detected in two countries [8, 9]. A prudent use of antibiotics is required to mitigate AMR development [10]. In relation to PWD, prudent use could entail a pig or pen based therapeutic treatment protocol, where identification of a bacterial agent is required before antibiotic treatment is started.

Presently, microbiological diagnostics of PWD include culture and/or PCR, which is expensive, time-consuming and laborious, and hence incompatible with pre-treatment diagnostics. Point-of-care tests (POCT) are diagnostic devices that within a short period can determine the causative agent at or near the point of care. The commercially available POCTs range from single pathogen lateral flow assays to multiplex real time polymerase chain reactions. POCTs for *Staphylococcus aureus* in man [11] and for mastitis in cattle [12] have been shown to significantly decrease antibiotic use by up to half.

A POCT for PWD would potentially make it possible to differentiate between bacterial and non-bacterial causes of PWD and therefore guide farmers and veterinarians in the decision on antibiotic treatment. A POCT, Rainbow Piglet Scours POCT (BIOK 374, Bio-X Diagnostics A.S., Rochefort, Belgium) is commercially available, but have, to the authors’ knowledge, not been evaluated. Therefore, this study aimed to evaluate the diagnostic performance of the Rainbow Piglet Scours test for PWD in two herds.

## Results

In both herd A and B the diarrhoea outbreak occurred 3 days after weaning. In herd A, faecal samples were obtained from 364 pigs, whereof 55 were scored as diarrhoea, equalling a prevalence of 15%. In herd B, 324 faecal samples were obtained and 150 were scored as diarrhoea giving a prevalence of 46%. All 55 diarrhoeic samples from herd A and 60 of the 150 diarrhoeic samples from herd B were selected for the study. For the selected faecal diarrhoeic samples (n = 115), 60 had faecal consistency score 3 and 55 had faecal consistency score 4. All selected faecal samples were tested using real-time PCR and the Rainbow Piglet Scours POCT. Due to non-valid results on the POCT, 110 samples were included in the diagnostic evaluation for ETEC F4, 112 samples for ETEC F18 and 115 samples for rotavirus.

### Results from the microbiological PCR analyses

**Fig. 1:**
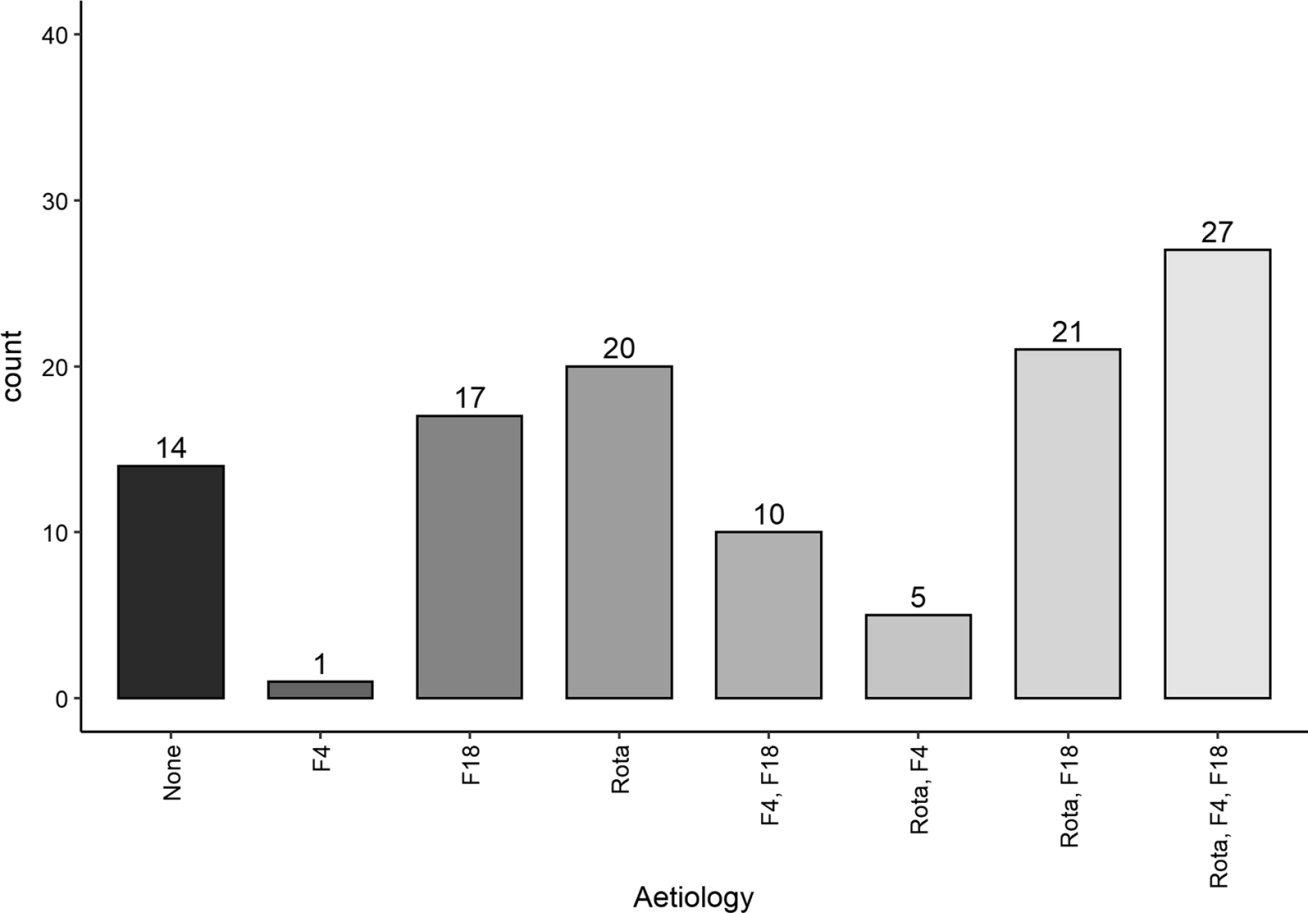
Pathogens detected in 115 diarrhoeic faecal samples by microbiological PCR analyses. F4 = ETEC F4, F18 = ETEC F18 and Rota = Rotavirus

Results from the microbiological PCR analyses demonstrated that out of the 115 diarrhoiec samples, 12.2% (n = 14) were negative for all three investigated pathogens, 33% (n = 38) were positive for one pathogen, 31.3% (n = 36) for two pathogens and 23.5% (n = 27) were positive for all three pathogens. The microbiological PCR results can be found in Fig. 1.

### Detection limits for the POCT

Comparison between the POCT and microbiological PCR analyses showed that the POCT was able to give a positive result up to a quantification cycle (C_q_) value of 21.1 for ETEC F4. For ETEC F18, the POCT was able to give a positive result up to a C_q_ value of 18.4, while for rotavirus it was up to a C_q_ value of 22.6.

### Diagnostic performance of the POCT

**Fig. 2:**
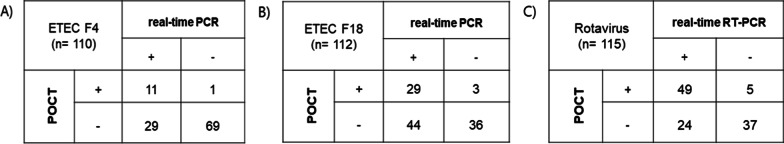
Cross-tabulations for the POCT (Rainbow Piglets Scours POCT) and the microbiological PCR analyses of diarrhoeic faecal samples. **A** ETEC F4, **B** ETEC F18 and **C** Rotavirus. + is positive faecal sample, − is negative faecal sample

The results from the POCT and the microbiological PCR analyses have been summarized in Fig. 2.

For all three pathogens, the POCT had a high level of false negatives and a low level of false positives, leading to a high diagnostic specificity but a low diagnostic sensitivity. The diagnostic sensitivity, specificity, positive predictive value (PPV) and negative predictive value (NPV) were calculated separately for the three pathogens, and has been summarized in Table 1 along with the kappa value. The Kappa values indicate a slight agreement between the POCT and the microbiological PCR analyses regarding ETEC F4 and ETEC F18 and a moderate agreement with rotavirus.

**Table 1:** Performance of the point-of-care test for ETEC F4, ETEC F18 and rotavirus

	Diagnostic Sensitivity	Diagnostic Specificity	Positive predictive value	Negative predictive value	Kappa
ETEC F4	0.28 (0.15–0.44)	0.99 (0.92–1.00)	0.92 (0.62–1.00)	0.70 (0.60–0.79)	0.31
ETEC F18	0.40 (0.28–0.52)	0.92 (0.79–0.98)	0.91 (0.75–0.98)	0.45 (0.34–0.56)	0.26
Rotavirus	0.67 (0.55–0.78)	0.88 (0.74–0.96)	0.91 (0.80–0.97)	0.61 (0.47–0.73)	0.50

Estimate (95% CI)

## Discussion

### Results and implications for the use of the evaluated POCT

The diagnostic sensitivity of the POCT was found to be 0.28, 0.40 and 0.67 and the NPV 0.70, 0.45 and 0.61 for ETEC F4, ETEC F18 and rotavirus, respectively. If the POCT was to be used as a pre-treatment diagnostic tool and as a guide to start antibiotic treatment on pig-level, the low sensitivity and negative predictive value for the *E. coli* subtypes would lead to a high level of false negatives and hence undertreatment. Using ETEC F18, which was the most prevalent ETEC subtype, as an example a negative test result on the POCT would only be correct in approximately 45% of the cases. However, the specificity and PPV was found to be relatively high, indicating a low level of false positives and a positive result for ETEC F18 would in 91% of cases be correct. A positive POCT result can therefore be trusted and such animals must be treated with antibiotics. However, due to the high level of false negatives and risk of substantial undertreatment, using the POCT to make diagnosis and treatment decisions for individual pigs cannot be recommended. For all three pathogens on the POCT, a high limit of detection (low analytical sensitivity) was established, demonstrating that only faecal samples containing a high number of F4, F18 and/or rotavirus would be positive on the POCT. The C_q_ values for POCT negative faecal samples (data not shown), revealed that the ETEC F4, ETEC F18 and rotavirus levels in the POCT false negative faecal samples were in fact below the limit of detection for all except one sample. This showed that the POCT false negative results can be explained by the low analytical sensitivity of the POCT.

Hence, to make the POCT more appropriate for individual diagnostics, the analytical sensitivity should be improved.

In the current study the focus was on test evaluation in the individual pig. Group evaluation would also be relevant, i.e. to evaluate the POCT for treatment decisions at herd, section or pen level. The POCT may be used to establish aetiology in a larger group of pigs if a representative number of pigs is tested, since a positive result would with high accuracy indicate that the aetiological agent is present in the tested group. Furthermore, using statistical formulae [13] the POCT could be used to estimate the true prevalence of an aetiological agent on herd or section level, from the apparent prevalence obtained from results using the POCT in a specific herd, section or pen.

POCT are diagnostic devices that within a short period can determine the causative agent at or near the point of care. POCT is a very broad term and it covers many very different laboratory techniques, including techniques used in a hospital and requires more heavy and/or expensive machinery. This should be taken into consideration when comparing evaluation studies and diagnostic performance measures of POCT.

Other studies evaluating the Rainbow Piglet Scours POCT were unfortunately not found, making a comparison of the found diagnostic performance difficult and more research should be focused on evaluating such commercial tests to ensure high accuracy and applicability in practice.

### Limitations of the study

The study should be considered preliminary work. One of the main limitations to our study is the amount of herds and batches included, since experience and experiments performed within our group (data unpublished) have shown large variation in aetiology and prevalence both between herds and between batches of pigs. The study included two conventional herds and one batch per herd and having included more herds and batches might have increased the representativeness of our study population to the target population. Another limitation is related to the prevalence of the examined infections. The prevalence has an impact on diagnostic performance, especially the positive and negative predictive value. So inclusion of a larger number of samples in different herds with different prevalence would have been beneficial.

## Conclusions

The Rainbow Piglet scours is a POCT that can be employed in a herd to determine the pathogens involved in a diarrhoea outbreak. However, due to low diagnostic sensitivity 28%, 40% and 67% for ETEC F4, ETEC F18 and rotavirus respectively, using it as a diagnostic tool for individual diagnosis of PWD would lead to undertreatment.

## Methods

### Sample size

The minimum sample size of 120 faecal samples, needed to validate the POCT, was determined based on testing if the agreement, between the reference standard and the POCT, was ≥ 80%, with a power of 80% and a 0.05 significance level [13]. It was decided to include two herds with 60 faecal samples each.

### Inclusion and diarrhoea outbreaks

A prospective study in two herds (Herd A and B) was conducted between November 23, 2020 and December 12, 2020. In total, one-week batch per herd was included. The herds weaned pigs without medicinal ZnO or systematically batch medication after weaning. Furthermore, the herds had to have recurring issues with PWD that was associated with an ETEC infection. Herd A, had a 14-day cycle and the pigs were weaned at approximately 35 days of age to a section containing 20 pens with 33 pigs per pen. The pigs had ad libitum access to water from a drinking nipple and were fed dry feed in a pen feeder. Herd B, had a one week cycle and the pigs were weaned at approximately 28 days of age to a section containing 10 pens with approximately 45 pigs in each pen. The pigs were fed dry feed in a pen feeder and had ad libitum access to water. From weaning and until an outbreak of diarrhoea occurred, the staff evaluated the level of diarrhoea in the section, daily.

### Experimental design

The procedure was identical for the two herds and was as following: When the herd employees observed the first outbreak of diarrhoea after weaning, the research team drove to the herd to collect faecal samples and perform tests for ETEC F4, ETEC F18 and rotavirus using the Rainbow Piglets Scours POCT (BIOK 374, Bio-X Diagnostics A.S., Rochefort, Belgium). Within the section experiencing outbreak of diarrhoea after weaning, faecal samples were collected from all pigs in the section by digital stimulation of the rectum and faecal consistency was scored from 1–4, using the method described by Pedersen and Toft, (2011) [14]. If the faecal sample was diarrhoeic (consistency score 3: Loose faeces or score 4: Watery faeces), the faecal sample was saved. After collection of faecal samples from all pigs within the section, 60 faecal samples with score 3–4 was chosen using simple randomization and used for validation of the POCT. From the 60 faecal samples a spoonful of faeces was used in the POCT and two eSwabs (SSI Diagnostica A/S, Hillerød, Denmark) were dipped into the sample. The eSwabs were placed in a sterile container containing Amies medium, stored in a cool place and analysed for ETEC F4, ETEC F18 and rotavirus A using PCR analyses.

### Point-of-care test

The POCT used was the Rainbow Piglet Scours BIOK374 (Bio-X Diagnostics A.S, Rochefort, Belgium). The POCT is antibody based and test was performed according to the manufacture’s instructions. In short, the test was performed by adding a small spoonful of faeces using the supplied spoon into the sample tube. Then the sample tube was shaken to ensure homogenization, and the sample tube was inserted into the strip tube. After securing the cap, the liquid from the sample tube was automatically released into the strip tube. The strip tube was placed vertically on a flat surface to allow migration up the test strips and results were noted after 10 min. The reference standard was performed after the POCT hence, the assessors were unaware of the results from the microbiological PCR analyses when performing the POCT. Similarly, lab technicians performing the microbiological PCR analyses were unaware of the results from the POCT.

### Laboratory analysis

#### Nucleic acid extraction

RNA and DNA were extracted from the eSwab samples using the extraction robot QIAcube HT (QIAGEN, Hilden, Germany) and the Cador Pathogen 96 QIAcube HT kit (QIAGEN) using the manufacturer’s instructions. Prior to nucleic acid extraction, the eSwab samples were prepared by vortexing the samples for 15 s followed by removal of the eSwabs from the medium. The samples were then centrifuged for 3 min at 5,500 × *g* at room temperature (15–25℃), and 200 µL of the supernatant was subsequently used for extraction. Positive and negative (nuclease-free water;

Amresco, Cleveland, OH) controls were included in each extraction. The nucleic acids were stored at − 80℃ until further analysis.

#### Microbiological PCR analyses

The eSwab samples were analysed for ETEC F4 and ETEC F18 (real-time PCR) and for rotavirus A (real-time RT-PCR) using the Rotor-Gene Q real-time platform (QIAGEN). For the real-time PCR assays targeting ETEC F4 and ETEC F18, JumpStart Taq Ready mix (Sigma-Aldrich, St. Louis, MO) was used with a final reaction volume of 25 µl. For ETEC F4, the PCR mix contained 12.5 µl JumpStart Taq ready mix (2*x*), 0.15 µl of each primer (100 µM), 0.05 µl probe (100 µM), 2 µl MgCl2 (25 mM), 7.15 µl nuclease-free water, and 3 µl DNA. For ETEC F18, the PCR mix contained 12.5 µl JumpStart Taq ready mix (2*x*), 0.15 µl of each primer (100 µM), 0.05 µl probe (100 µM), 3.5 µl MgCl2 (25 mM), 5.65 µl nuclease-free water, and 3 µl DNA. The PCR reactions were tested at the following thermal cycle conditions: 94℃ for 2 min, followed by 40 cycles of 94℃ for 15 s, and 60℃ for 60 s. Primer and probes sequences have been published elsewhere [15].

For the the real-time RT-PCR assay targeting rotavirus A, AgPath-ID one-step RT-PCR reagents kit (Applied Biosystems, Foster City, CA) was used with a final reaction volume of 15 µl. The PCR mix consisted of 7.5 µl RT-PCR buffer (2*x*), 0.12 µl of each primer (50 µM), 0.04 µl probe (50 µM), 0.6 µl RT-PCR enzyme mix (25*x*), 4.62 µl nuclease-free water, and 2 µl RNA. The PCR reactions were run at the following thermal cycling conditions: 45℃ for 10 min, 95℃ for 10 min, followed by 45 cycles of 94℃ for 15 s, and 60℃ for 45 s. Primer and probes sequences have been published elsewhere [16].

In each real-time PCR and real-time RT-PCR positive and negative (nuclease-free water;Amresco, Cleveland, OH) controls were run, and all reactions (samples, positive and negative controls) were run in duplicates.

#### Detection limits for the POCT

To determine the detection limits of the POCT for the ETEC F4, ETEC F18 and rotavirus analyses, two-fold serial dilutions (1–1:512) for the three pathogens were made and tested by the POCT and microbiological PCR analyses. The two-fold serial dilutions were made in 10% liquid negative faeces solution. For the POCT, 500 µL of each of the dilutions were used as test material and test strips specific for ETEC F4, ETEC F18 and rotavirus, respectively, were added to these tubes. The tubes were placed vertically on a flat surface to allow migration up the test strips and results were noted after 10 min, using the supplied instructions.

### Statistical analysis

The statistical analyses were performed using R version 4.0.2. A sample was defined as being positive in the POCT when both the control line and the test line was visible on the strip, and as negative when only the control line was visible. If no lines were visible the test was deemed invalid. For the real-time PCR analyses the quantification cycle was used to dichotomize the results into negative (C_q_ = 0) and positive (C_q_ > 0). The diagnostic performance, PPV and NPV were calculated based on 2 × 2 tables for each pathogen and the agreement between the two methods were evaluated using Cohen’s Kappa value.

## Data Availability

The datasets used and analysed during the current study are available from the corresponding author on reasonable request.
